# Concordance of Disclosed Hospital Prices With Total Reimbursements for Hospital-Based Care Among Commercially Insured Patients in the US

**DOI:** 10.1001/jamanetworkopen.2021.37390

**Published:** 2021-12-13

**Authors:** Michal Horný, Paul R. Shafer, Stacie B. Dusetzina

**Affiliations:** 1 Department of Radiology and Imaging Sciences, Emory University School of Medicine, Atlanta, Georgia; 2 Department of Health Policy and Management, Emory University Rollins School of Public Health, Atlanta, Georgia; 3 Department of Health Law, Policy, and Management, Boston University School of Public Health, Boston, Massachusetts; 4 Department of Health Policy, Vanderbilt University School of Medicine, Nashville, Tennessee; 5 Vanderbilt-Ingram Cancer Center, Nashville, Tennessee

## Abstract

**Importance:**

To improve health care price transparency and promote cost-conscious selection of health care organizations and practitioners, the Centers for Medicare & Medicaid Services (CMS) required that hospitals share payer-specific negotiated prices for selected shoppable health services by January 2021. While this regulation improves price transparency, it is unclear whether disclosed prices reflect total costs of care, since many hospital-based services are delivered and billed separately by independent practitioners or other health care entities.

**Objective:**

To assess the extent to which prices disclosed under the new hospital price transparency regulation are correlated with total costs of care among commercially insured individuals.

**Design, Setting, and Participants:**

This cross-sectional study used a large database of commercial claims from 2018 to analyze encounters at US hospitals for shoppable health care services for which price disclosure is required by CMS. Data were analyzed from November 2020 to February 2021.

**Exposures:**

Whether the service was billed by the hospital or another entity.

**Main Outcomes and Measures:**

Outcomes of interest were the percentage of encounters with at least 1 service billed by an entity other than the hospital providing care, number of billing entities, amounts billed by nonhospital entities, and the correlation between hospital and nonhospital reimbursements.

**Results:**

The study analyzed 4 545 809 encounters for shoppable care. Independent health care entities were involved in 7.6% (95% CI, 6.7% to 8.4%) to 42.4% (95% CI, 39.1% to 45.6%) of evaluation and management encounters, 15.9% (95% CI, 15.8% to 16%) to 22.2% (95% CI, 22% to 22.4%) of laboratory and pathology services, 64.9% (95% CI, 64.2% to 65.7%) to 87.2% (95% CI, 87.1% to 87.3%) of radiology services, and more than 80% of most medicine and surgery services. The median (IQR) reimbursement of independent practitioners ranged from $61 ($52-$102) to $412 ($331-$466) for evaluation and management, $5 ($4-$6) to $7 ($4-$12) for laboratory and pathology, $26 ($20-$32) to $210 ($170-$268) for radiology, and $47 ($21-$103) to $9545 ($7750-$18 277) for medicine and surgery. The reimbursement for services billed by the hospital was not strongly correlated with the reimbursement of independent clinicians, ranging from *r* = −0.11 (95% CI, −0.69 to 0.56) to *r* = 0.53 (95% CI, 0.13 to 0.78).

**Conclusions and Relevance:**

This cross-sectional study found that independent practitioners were frequently involved in the delivery of shoppable hospital-based care, and their reimbursement may have represented a substantial portion of total costs of care. These findings suggest that disclosed hospital reimbursement was usually not correlated with total cost of care, limiting the potential benefits of the hospital price transparency rule for improving consumer decision-making.

## Key Points

**Question:**

Are prices of hospital services disclosed under the new federal hospital price transparency rule correlated with total costs of hospital-based care among commercially insured individuals?

**Findings:**

In this cross-sectional study of 4 545 809 hospital-based encounters for shoppable care in 2018, health care entities that billed for their services independently from the hospital were frequently involved in care delivery, and their reimbursements comprised substantial portions of the total costs of care. The disclosed hospital prices were weakly correlated with the reimbursements of independent health care entities.

**Meaning:**

These findings suggest that prices disclosed under the new regulation may have limited value to patients attempting to make cost-conscious health care choices.

## Teaser

This cross-sectional study assesses the likelihood of correlation between prices disclosed under the new Centers for Medicare & Medicaid Services hospital price sharing regulation and total costs of care among commercially insured individuals.

## Introduction

Price transparency in health care is promoted as a means of empowering patients to become active consumers who can make informed decisions regarding practitioner and treatment options. However, obtaining meaningful price information before receiving care has historically been difficult for patients, and existing price transparency tools have struggled to gain traction.^1,2,3,4,5,6,7,8,9,10,11,12^ To improve health care price discovery, the Centers for Medicare & Medicaid Services (CMS) issued a rule requiring that hospitals share payer-specific negotiated prices for selected shoppable health care services defined by CMS as services that “can be scheduled by a health care consumer in advance,” by January 2021.^13^

While this regulation improves health care price transparency to a degree, patients seeking care may still be unable to obtain accurate estimates of total costs in advance of their care, as many services integral to hospital-based care are delivered and billed separately by independent practitioners or health care entities who are not subject to this regulation. The objective of this study is to assess the extent to which disclosed prices under the new hospital price transparency rule are correlated with total costs of care among commercially insured individuals.

## Methods

This cross-sectional study was deemed exempt from institutional review board review and informed consent by Emory University because we used deidentified data. This study followed the Strengthening the Reporting of Observational Studies in Epidemiology (STROBE) reporting guideline for cross-sectional studies.

The CMS hospital price transparency rule requires hospitals operating in the United States to publicly share payer-specific negotiated prices for at least 300 shoppable health care services, 70 of which are specified by the regulation.^13^ This study focuses on these 70 services, which fall into 4 broad categories: evaluation and management, laboratory and pathology, radiology, and medicine and surgery. Services specified by a Diagnosis-Related Group code were assumed to be delivered in the inpatient setting. The appropriate setting of care delivery (inpatient or outpatient) for services specified by Current Procedural Terminology (CPT) or Healthcare Common Procedures Coding System codes was determined based on typical patterns of care. Additionally, we identified a set of common ancillary services for which patients could be billed in connection with provision of each shoppable service (eTable 1 in the Supplement). The identified care delivery settings and lists of potential ancillary services were reviewed for accuracy by clinical experts within relevant medical subspecialties.

We extracted health care entity reimbursements from the 2018 MarketScan Commercial Database (IBM), one of few national multi-insurer commercial health insurance claims databases providing a large sample of claims for individuals covered by employer-sponsored plans across the United States, which cover the preponderance of the commercially insured population in the US. The 2018 data include 27 087 740 enrolled individuals aged 0 to 64 years from all 50 states, the District of Columbia, and Puerto Rico. To assemble service packages for which patients were billed, we first identified hospital-based encounters for shoppable care using relevant CPT, Healthcare Common Procedures Coding System, or Diagnosis-Related Group codes for the 70 CMS-specified services. Then, we extracted claims submitted for patients on the same day (for outpatient encounters) or during the same hospital stay (for inpatient encounters) that satisfied the ancillary-service inclusion criteria, as described in eTable 1 in the Supplement. To avoid capturing encounters not suited for price shopping, we excluded encounters associated with claims indicating complex situations (eg, prostatectomy performed as part of cystectomy, unsuccessful vaginal delivery completed by cesarean birth), inpatient encounters indicating the presence of complicating diagnoses (unless explicitly requested by the CMS rule), and all encounters associated with claims from the emergency department, urgent care, or ambulance services. The package-specific exclusion criteria are described in greater detail in eTable 1 in the Supplement. Finally, we excluded encounters that involved capitated services and those performed at out-of-network hospitals, as we would be unable to observe full prices of these services. Flowcharts of sample inclusion and exclusion criteria overall and by service are presented in eFigure 1 and eTable 2 in the Supplement.

### Statistical Analysis

For each service package, we identified health care entities that billed for care using anonymized taxpayer identification numbers included on individual claims and calculated their reimbursement. We used 4 metrics to assess the extent to which disclosed prices under the new hospital price transparency rule were likely to reflect total costs of care. First, we calculated the percentage of encounters that involved at least 1 service billed by an entity other than the hospital. This outcome measures the prevalence of billing for a single episode of care by multiple health care entities, which inherently means that disclosure of hospital prices gives patients an incomplete estimate of the total costs of their care. Second, to examine how many entities a patient would have to contact to obtain an estimate of the total costs of care, we assessed the number of billing entities other than the hospital involved in care delivery. Third, we assessed the reimbursement amounts billed by nonhospital entities, which the CMS regulation does not require to be publicly disclosed. We summarized the findings in terms of absolute dollar amounts and as percentages of the publicly disclosed hospital reimbursements. Finally, to evaluate whether the disclosed price for the hospital-billed services is correlated with the nondisclosed cost of care by independent health care entities, we calculated the Pearson correlation coefficients between the hospital and nonhospital reimbursement components. Statistical significance for correlation coefficients is indicated by 95% CIs that do not cross 0. The analysis was completed using SAS statistical software version 9.4 (SAS Institute). Data were analyzed from November 2020 to February 2021.

## Results

The final sample consisted of 4 545 809 hospital encounters with shoppable service packages. The sample size by service type is reported in eTable 2 in the Supplement. For the 70 shoppable services, the chance of receiving care at a hospital from health care entities that billed for care separately from the hospital varied widely by service (Table). Independent entities were involved in delivery of 7.6% (95% CI, 6.7% to 8.4%) to 42.4% (95% CI, 39.1% to 45.6%) of evaluation and management services, with median (IQR) reimbursement for these entities ranging from $61 ($52-$102) for a 45-minute psychotherapy session to $412 ($331-$466) for a 60-minute office consultation. Among laboratory and pathology services, 15.9% (95% CI, 15.8% to 16%) to 22.2% (95% CI, 22% to 22.4%) of encounters involved independent entities, with relatively low median (IQR) reimbursement ranging from $5 ($4-$6) to $7 ($4-$12). Independent radiologists provided their services in 64.9% (95% CI, 64.2% to 65.7%) to 87.2% (95% CI, 87.1% to 87.3%) of imaging services, with median (IQR) reimbursement ranging from $26 ($20-$32) for lower back radiography to $210 ($170-$268) for brain magnetic resonance imaging. Involvement of independent entities was generally most common and most costly in complex medicine and surgery services. Independent entities were involved in more than 80% of each medicine and surgery shoppable service, except for physical therapy (0.3%; 95% CI, 0.3% to 0.4%), routine electrocardiogram (0.7%; 95% CI, 0.3% to 1.1%), left heart catheterization (53.3%; 95% CI, 38.8% to 67.9%), and sleep study (59.2%; 95% CI, 58.4% to 60.1%). The median (IQR) reimbursement for independent entity services ranged from $47 ($21-$103) for physical therapy to $9545 ($7750-$18 277) for a major cardiothoracic procedure.

**Table.** Involvement of Entities That Billed Separately From Hospitals in Hospital-Based Care Delivery and the Reimbursement for Their Services, by Type of Service

Shoppable service	≥1 service in package billed by nonhospital entity, % of service packages (95% CI)	Median (IQR)			Correlation between reimbursement for services billed by hospital and those billed by nonhospital entities, conditional, *r* (95% CI)
		Nonhospital entities that billed for care, conditional, No.	Reimbursement for services billed by nonhospital entities, conditional, $	Reimbursement for services billed by nonhospital entities, conditional, % of hospital reimbursement	
**Evaluation and management**					
Psychotherapy, session length, min					
30	10.8 (10.1 to 11.5)	1 (1 to 1)	63 (46 to 111)	72 (49 to 111)	0.06 (−0.01 to 0.13)
45	9.9 (9.6 to 10.2)	1 (1 to 1)	61 (52 to 102)	63 (47 to 82)	0.1 (0.07 to 0.13)
60	32.1 (31.3 to 32.8)	1 (1 to 1)	77 (75 to 107)	71 (63 to 92)	0.27 (0.24 to 0.3)
Family psychotherapy, 50 min					
Not including patient	9.3 (7.4 to 11.3)	1 (1 to 1)	109 (86 to 140)	49 (41 to 101)	0.14 (−0.09 to 0.35)
Including patient	7.6 (6.7 to 8.4)	1 (1 to 1)	117 (77 to 166)	78 (48 to 120)	0.28 (0.17 to 0.38)
Group psychotherapy	8.9 (8.7 to 9.1)	1 (1 to 1)	85 (57 to 150)	36 (19 to 75)	−0.01 (−0.03 to 0.02)
New patient office or other outpatient visit, typical session length, min					
30	12.4 (11.8 to 13.0)	1 (1 to 1)	118 (90 to 164)	81 (41 to 159)	0.04 (−0.01 to 0.09)
45	16.5 (15.7 to 17.3)	1 (1 to 1)	229 (162 to 285)	111 (60 to 214)	−0.01 (−0.07 to 0.04)
60	23.1 (21.8 to 24.5)	1 (1 to 1)	298 (178 to 389)	84 (50 to 152)	−0.03 (−0.09 to 0.04)
Patient office consultation, typical session length, min					
40	16.4 (14.3 to 18.5)	1 (1 to 1)	264 (139 to 295)	146 (92 to 228)	0.07 (−0.07 to 0.21)
60	32.6 (30.1 to 35.1)	1 (1 to 1)	412 (331 to 466)	183 (131 to 357)	−0.1 (−0.19 to −0.01)
Initial new patient preventive medicine evaluation, patient age, y					
18-39	42.4 (39.1 to 45.6)	1 (1 to 1)	182 (138 to 226)	70 (32 to 137)	0.11 (0.01 to 0.21)
40-64	34.2 (30.4 to 38.0)	1 (1 to 1)	226 (155 to 275)	78 (42 to 139)	0.12 (−0.01 to 0.26)
**Laboratory and pathology**					
Basic metabolic panel	18.0 (17.9 to 18.2)	1 (1 to 1)	6 (4 to 12)	11 (5 to 26)	0.08 (0.07 to 0.09)
Blood test, comprehensive group of blood chemicals	18.1 (18.0 to 18.2)	1 (1 to 1)	5 (4 to 12)	11 (5 to 23)	0.03 (0.02 to 0.04)
Obstetric blood test panel	17.2 (15.5 to 18.9)	1 (1 to 1)	6 (4 to 10)	4 (2 to 11)	0.03 (−0.07 to 0.14)
Blood test, lipids (cholesterol and triglycerides)	20.5 (20.4 to 20.7)	1 (1 to 1)	5 (4 to 13)	13 (6 to 25)	0.05 (0.04 to 0.05)
Kidney function panel test	15.4 (14.7 to 16.1)	1 (1 to 1)	5 (4 to 10)	6 (3 to 17)	0.01 (−0.04 to 0.05)
Liver function blood test panel	18.0 (17.9 to 18.2)	1 (1 to 1)	6 (4 to 13)	13 (6 to 32)	0.12 (0.10 to 0.14)
Manual urinalysis test with examination using microscope	18.1 (18.0 to 18.2)	1 (1 to 1)	6 (4 to 8)	21 (9 to 64)	0.02 (0.01 to 0.04)
Automated urinalysis test	17.2 (15.5 to 18.9)	1 (1 to 1)	5 (4 to 6)	24 (9 to 66)	0.04 (0.02 to 0.06)
PSA	20.5 (20.4 to 20.7)	1 (1 to 1)	5 (4 to 11)	10 (6 to 19)	0.04 (0.02 to 0.05)
Blood test, TSH	15.4 (14.7 to 16.1)	1 (1 to 1)	5 (4 to 13)	12 (6 to 23)	0.04 (0.04 to 0.05)
Complete blood cell count, with differential white blood cells, automated	15.9 (15.8 to 16.0)	1 (1 to 1)	6 (4 to 9)	15 (7 to 35)	0.03 (0.03 to 0.04)
Complete blood count, automated	22.2 (22.0 to 22.4)	1 (1 to 1)	7 (4 to 12)	16 (9 to 34)	0.11 (0.10 to 0.13)
Blood test, clotting time	18.8 (18.5 to 19.1)	1 (1 to 1)	6 (4 to 7)	12 (6 to 32)	0.1 (0.08 to 0.11)
Coagulation assessment blood test	20.2 (19.8 to 20.7)	1 (1 to 1)	6 (5 to 9)	10 (5 to 27)	0.09 (0.07 to 0.11)
**Radiology**					
CT scan, head or brain, without contrast	81.7 (81.0 to 82.5)	1 (1 to 1)	76 (60 to 92)	13 (7 to 21)	0.16 (0.14 to 0.18)
MRI scan of brain before and after contrast	83.8 (83.4 to 84.1)	1 (1 to 1)	210 (170 to 268)	14 (9 to 22)	0.33 (0.32 to 0.34)
Radiograph, lower back, minimum four views	76.0 (75.4 to 76.7)	1 (1 to 1)	26 (20 to 32)	10 (6 to 18)	0.18 (0.17 to 0.20)
MRI scan of lower spinal canal	84.0 (83.6 to 84.4)	1 (1 to 1)	133 (107 to 166)	13 (8 to 20)	0.34 (0.33 to 0.35)
CT scan, pelvis, with contrast	78.4 (75.6 to 81.2)	1 (1 to 1)	105 (85 to 126)	12 (7 to 18)	0.10 (0.02 to 0.18)
MRI scan of leg joint	81.5 (81.1 to 82.0)	1 (1 to 1)	125 (98 to 152)	12 (8 to 18)	0.25 (0.24 to 0.26)
CT scan of abdomen and pelvis with contrast	84.3 (84.0 to 84.7)	1 (1 to 1)	166 (126 to 204)	13 (7 to 23)	0.07 (0.06 to 0.08)
Ultrasonography of abdomen	81.7 (81.3 to 82.1)	1 (1 to 1)	69 (54 to 85)	19 (11 to 27)	0.11 (0.10 to 0.12)
Abdominal ultrasonography of pregnant uterus (≥14 wk, 0 d) single or first fetus	64.9 (64.2 to 65.7)	1 (1 to 1)	86 (68 to 103)	26 (16 to 35)	0.12 (0.10 to 0.13)
Ultrasonography of pelvis through vagina	76.3 (75.9 to 76.7)	1 (1 to 1)	59 (48 to 71)	20 (13 to 29)	0.21 (0.20 to 0.22)
Mammography					
1 breast	85.5 (85.3 to 85.8)	1 (1 to 1)	63 (48 to 81)	29 (18 to 46)	0.04 (0.03 to 0.05)
Both breasts	86 (85.7 to 86.3)	1 (1 to 1)	77 (58 to 100)	30 (20 to 49)	0.09 (0.08 to 0.10)
Screening, bilateral	87.2 (87.1 to 87.3)	1 (1 to 1)	57 (45 to 75)	29 (20 to 44)	0.05 (0.04 to 0.05)
**Medicine and surgery**					
Cardiac valve and other major cardiothoracic procedures with cardiac catheterization with major complications or comorbidities	100 (100 to 100)	5.5 (2 to 7)	9546 (7750 to 18 277)	10 (9 to 16)	0.53 (0.13 to 0.78)
Spinal fusion except cervical^a^	96.1 (95.3 to 97.0)	4 (2 to 5)	8968 (6495 to 12 999)	17 (11 to 25)	0.19 (0.15 to 0.23)
Major joint replacement or reattachment of lower extremity^a^	95.1 (94.7 to 95.4)	3 (2 to 4)	3910 (3016 to 5005)	14 (9 to 19)	0.20 (0.18 to 0.22)
Cervical spinal fusion^a^	95.3 (93.8 to 96.9)	3 (2 to 4)	7525 (5214 to 10 708)	26 (15 to 42)	0.17 (0.10 to 0.24)
Uterine and adnexa procedures for nonmalignant neoplasm^a^	91.8 (90.8 to 92.9)	3 (2 to 3)	2586 (1687 to 3647)	19 (12 to 29)	0.13 (0.09 to 0.17)
Removal of ≥1 breast growth, open procedure	94.8 (93.7 to 95.9)	3 (2 to 3)	1165 (816 to 1564)	25 (14 to 41)	0.18 (0.14 to 0.23)
Shaving of shoulder bone using an endoscope	93.0 (92.3 to 93.8)	2 (2 to 3)	3473 (2485 to 4742)	34 (20 to 54)	0.17 (0.14 to 0.20)
Removal of 1 knee cartilage using an endoscope	91.7 (91.1 to 92.3)	2 (2 to 2)	1433 (1075 to 1822)	27 (16 to 43)	0.15 (0.13 to 0.18)
Removal of tonsils and adenoid glands patient aged <12 y	90.7 (89.8 to 91.6)	2 (2 to 3)	1087 (851 to 1375)	23 (14 to 37)	0.02 (−0.02 to 0.05)
Endoscopic procedure					
Diagnostic examination of esophagus, stomach, and/or upper small bowel	86.1 (85.2 to 86.9)	1 (1 to 2)	464 (211 to 768)	25 (12 to 45)	0.14 (0.11 to 0.16)
Biopsy of the esophagus, stomach, and/or upper small bowel	92.6 (92.4 to 92.8)	2 (1 to 3)	712 (385 to 1105)	31 (16 to 55)	0.16 (0.15 to 0.17)
Diagnostic examination of large bowel	86.4 (85.9 to 86.8)	2 (1 to 2)	670 (411 to 958)	33 (19 to 54)	0.12 (0.11 to 0.13)
Biopsy of large bowel	92.6 (92.4 to 92.9)	2 (2 to 3)	903 (591 to 1310)	40 (23 to 64)	0.18 (0.17 to 0.19)
Removal of polyps or growths of large bowel	93.2 (92.9 to 93.5)	2 (2 to 3)	957 (661 to 1321)	45 (26 to 72)	0.11 (0.10 to 0.12)
Ultrasonographic examination of lower large bowel	88.4 (78.8 to 98.0)	1.5 (1 to 2)	864 (615 to 1164)	39 (23 to 49)	0.14 (−0.19 to 0.44)
Removal of gallbladder	94.0 (93.5 to 94.5)	3 (2 to 3)	1953 (1561 to 2475)	24 (16 to 38)	0.04 (0.02 to 0.06)
Repair of groin hernia patient age ≥5 y	90.1 (89.0 to 91.3)	2 (1 to 2)	1470 (1110 to 1914)	25 (15 to 40)	0.03 (−0.01 to 0.07)
Biopsy of prostate gland	85.6 (83.7 to 87.5)	2 (1 to 3)	1130 (735 to 1831)	35 (19 to 63)	0.16 (0.10 to 0.21)
Surgical removal of prostate and surrounding lymph nodes using an endoscope	95.7 (94.5 to 96.9)	2 (1 to 3)	4830 (3778 to 6335)	28 (19 to 43)	0.27 (0.21 to 0.33)
Routine obstetric care, including predelivery and postdelivery care					
For vaginal delivery	95.2 (95.0 to 95.5)	2 (2 to 3)	3965 (3153 to 5062)	53 (38 to 73)	0.24 (0.23 to 0.25)
For cesarean delivery	97.2 (96.9 to 97.5)	3 (2 to 3)	4580 (3675 to 5783)	43 (32 to 58)	0.21 (0.19 to 0.22)
For vaginal delivery after prior cesarean delivery	93.4 (91.7 to 95.1)	2 (1 to 2)	3985 (3176 to 5129)	55 (39 to 75)	0.19 (0.12 to 0.26)
Injection of substance into spinal canal of lower back or sacrum using imaging guidance	81.1 (80.3 to 82.0)	1 (1 to 1)	184 (131 to 325)	15 (9 to 31)	0.01 (−0.01 to 0.04)
Injections of anesthetic and/or steroid drug into lower or sacral spine nerve root using imaging guidance	85.7 (85.0 to 86.5)	1 (1 to 1)	238 (169 to 455)	21 (12 to 40)	0.19 (0.17 to 0.22)
Removal of recurring cataract in lens capsule using laser	84.5 (81.1 to 87.9)	1 (1 to 1)	418 (315 to 555)	49 (26 to 83)	−0.004 (−0.11 to 0.10)
Removal of cataract with insertion of lens	93.1 (92.5 to 93.8)	2 (1 to 2)	1291 (1018 to 1672)	31 (20 to 47)	0.18 (0.15 to 0.21)
Electrocardiogram, routine, with interpretation and report	0.7 (0.3 to 1.1)	1 (1 to 1)	38 (16 to 53)	11 (7 to 39)	−0.11 (−0.69 to 0.56)
Insertion of catheter into left heart for diagnosis	53.3 (38.8 to 67.9)	1 (1 to 1)	64 (26 to 278)	1 (0 to 5)	0.24 (−0.18 to 0.59)
Sleep study	59.2 (58.4 to 60.1)	1 (1 to 1)	200 (145 to 255)	9 (6 to 13)	0.13 (0.11 to 0.15)
Physical therapy, therapeutic exercise	0.3 (0.3 to 0.4)	1 (1 to 1)	47 (21 to 103)	25 (9 to 55)	0.17 (0.14 to 0.20)

Abbreviations: CT, computed tomography; MRI, magnetic resonance imaging; PSA, prostate specific antigen; TSH, thyroid stimulating hormone.

^a^ Without major comorbid conditions or complications.

The number of entities involved in care generally increased with service package complexity (Table). Among service packages that included independent entities, the highest median (IQR) number of nonhospital billing entities involved in the encounter was 5.5 (2-7) entities for a major cardiothoracic procedure and 1 (1-1) entity for all evaluation and management, laboratory and pathology, and radiology services.

When entities that billed for care independently from the hospital were involved in care delivery, their aggregate reimbursement both in dollars and as a percentage of the hospital portion of costs was often nontrivial (Table). For example, 95.2% of routine obstetric services for vaginal delivery involved care from entities that billed separately from the hospital. In those encounters, the median (IQR) reimbursement for services billed by the hospital was $7678 ($5744-$10 301), while the median (IQR) reimbursement for services billed by independent entities was $3965 ($3153-$5062).

We also found that the reimbursement amount for services billed by the hospital, ie, the price that would be disclosed under the CMS hospital price transparency rule, was not strongly correlated with the reimbursement of independent entities (Table; eFigure 2 in the Supplement). The correlation between the 2 components of the total reimbursement was generally low, ranging from *r* = −0.11 (95% CI, −0.69 to 0.56) for routine electrocardiogram to *r* = 0.53 (95% CI, 0.13 to 0.78) for a major cardiothoracic procedure.

## Discussion

This cross-sectional study found that practitioners who billed for care independently were frequently involved in the delivery of shoppable hospital-based care, and their reimbursement represented a substantial portion of the total cost of care. Many health care price transparency initiatives and tools have been developed to encourage patients to shop for more cost-effective care and decrease health care spending but have yielded low awareness, offered limited information, and had little evidence that they meaningfully influenced patient behavior.^2,3,4,5,6,7,9,10,11^ For example, some state-level price transparency tools have focused on publishing within-hospital mean prices, but these aggregated prices are not necessarily relevant to individual patients.^2^ Other initiatives required hospitals to publish charges, but those amounts rarely reflect the actual costs of care or potential cost-sharing responsibility for insured patients.^10^ Moreover, most price transparency tools offer price estimates for individual health care services (eg, a single procedure code), but not prices for entire episodes of care.^2,10^ Owing to these limitations and poor communication about them to patients, uptake of price transparency tools has been low^3,4,6,7^ and their availability has not been associated with lower health care spending.^5,6,11,14^

The CMS hospital price transparency rule addresses some of these challenges. Specifically, the regulation requires hospitals to publicly disclose plan-specific negotiated rates, including packages of ancillary services that are often provided by the hospital in conjunction to the main shoppable service. Nevertheless, aside from poor hospital adherence to the new requirements,^15^ the regulation still leaves several important gaps that limit patients’ ability to engage in meaningful price discovery. First, the regulation applies only to hospitals, while many shoppable health care services can be provided in other care delivery settings (eg, physicians’ offices, ambulatory surgical centers, independent imaging centers).^16,17,18^ Patients may still be unable to obtain prices of care in those settings. Second, the regulation requires price disclosure for a limited number of health care services. Patients seeking other types of care may still be unable to obtain useful price estimates. Third, the regulation requires price disclosure for 300 shoppable services, 70 of which are specified by the regulation, while selection of the remaining 230 services is left to individual hospitals at their discretion. Thus, for some health care services, patients may find prices at some local hospitals but not others, limiting their ability to make an informed choice. Finally, prices of shoppable service packages reported by hospitals do not include prices of integral services delivered by practitioners who are not hospital employees. Our analysis highlights just how problematic this final issue can be for price transparency, severely limiting the value to patients of the disclosures required under the CMS hospital price transparency rule. Specifically, this study documents how often independent entities were involved in delivery of select shoppable hospital-based services, the amount of total reimbursements that would not be publicly disclosed under the CMS hospital price transparency rule, and whether disclosed hospital prices were correlated with this undisclosed portion of the total reimbursements.

The root cause of incomplete price transparency is fragmentation of health care delivery and billing. A single hospital-based health care encounter from the patient’s perspective often consists of multiple services, some of which are provided by practitioners who are hospital employees, while others are provided by practitioners who deliver care at the hospital facility but contract with payers independently. Our study documents that independent entities were frequently involved in hospital-based care delivery. Their involvement was most common in medicine and surgery services and radiology services, less so in evaluation and management and laboratory and pathology services. The additional cost of independent practitioner reimbursement may create an unexpected and considerable financial burden for patients. While the median reimbursement of independent entities for laboratory and pathology services was low ($5-$7, depending on service type), it amounted to hundreds or thousands of dollars for most medicine and surgery services (eg, $903 for large bowel biopsy, $3965 for routine obstetric care for vaginal delivery).

Providing patients with incomplete price estimates may not be problematic if they seek to undergo an expensive procedure at an in-network hospital and are guaranteed that no out-of-network entities will be involved in care delivery. Because hospital services tend to be costly and federal regulations impose limits on patients’ annual out-of-pocket spending on in-network care, patients may reach their annual out-of-pocket maximums with the portion of costs billed by the hospital alone, leaving the remaining undisclosed costs of services from independent in-network entities to health plans. However, involvement of out-of-network entities in care delivered at in-network hospitals is relatively common.^19,20,21^ Because out-of-network bills can be quite high, this troublesome market failure also stemming from the fragmentation of health care delivery has been addressed by legislative actions on the state and federal levels.^22^

As US health care delivery is becoming increasingly vertically integrated,^23,24,25^ the chance of receiving care from entities that bill separately from the hospital is likely to decrease. While this trend can eventually lead to improved price transparency, vertical integration also leads to increased prices and spending for outpatient care.^26,27^ Nevertheless, our findings demonstrate that independent entities were frequently involved in hospital-based care delivery, and their reimbursement, the undisclosed component of the total cost of care, could be meaningful to patients, especially to those enrolled in high-deductible health plans who seek to undergo a procedure with total practitioner reimbursement that is lower than their annual deductible or out-of-pocket maximum.

Given the fragmented health care system, are there opportunities to improve on existing price transparency efforts to provide more comprehensive and accurate estimates of the total costs of care for patients? As long as patient cost-sharing is a function of practitioner reimbursement (eg, coinsurance, deductibles), all health care entities involved in care delivery should be subject to price transparency requirements. Obtaining prices of health care, and especially out-of-pocket cost estimates for insured patients is most practical from insurers, not care entities.^28^ Insurance companies have most of the necessary information needed to estimate individual patients’ out-of-pocket costs for nearly any service at their disposal, such as negotiated rates for individual health care services, lists of health care facilities and practitioners available in relevant health care markets, as well as information on patients’ plan benefit design, including amounts that patients already paid toward their annual deductibles and out-of-pocket maximums.

Efforts to obtain out-of-pocket cost estimates from health plans have already been introduced. The Transparency in Coverage rule,^29^ which will become partially effective in 2023 and fully effective in 2024, will require non-grandfathered commercial health plans to provide patients with personalized estimates of out-of-pocket costs for individual health care services by all types of in-network health care entities. However, even this regulation has notable limitations. First, the regulation will not improve health care price discovery for individuals enrolled in grandfathered plans or uninsured individuals. Additional efforts will be needed to achieve health care price transparency for all. Second, patients seeking to obtain an accurate estimate of their out-of-pocket costs will be expected to anticipate what individual services will constitute the planned health care encounter. This would be a challenging task for experienced health care experts, let alone nonexpert patients. As such, patients may still receive partial price information even under this prospective regulation. Insurers could take the lead on creating reliable packages of services across entities involved in episodes of care to provide their enrollees with useful price information before receiving care. Moreover, employers may demand such a service from insurers as a means of cost containment.

### Limitations

This study has some limitations. The composition of service packages likely varies not only across hospitals, but also within hospitals, depending on contracts between payers and health care entities, and patient-specific circumstances. We constructed service packages using applicable claims that were billed to patients on the same day or during the inpatient stay as the main shoppable service. However, some health systems may also include services provided to the patient before or after the main service (eg, preoperative and postoperative care) in their disclosed price estimates, and in such cases, the reimbursement amounts for services billed by the hospital and those billed by entities other than hospital would be higher than our estimates. Additionally, the set of shoppable evaluation and management services specified in the CMS rule includes various durations of new patient office or other outpatient visit (30, 45, and 60 minutes; CPT codes 99203-99205) and patient office consultation (40 and 60 minutes; CPT codes 99243-99244).^13^ Claims with these codes are commonly billed by practitioners in a broad range of clinical specialties for outpatient visits to address a variety of health care needs. Because the lists of potential ancillary services depend on the reasons for each visit or consultation, for these broadly applicable evaluation and management services, we constructed only minimal service packages. As such, our quantifications of the associated hospital and nonhospital reimbursements for these services are likely underestimated. Additionally, we used a large national convenience sample of commercial insurance claims to perform this analysis, and as such, our findings may not generalize to hospitals or insurers not included. However, given the large size of the database, we are confident in our ability to draw broad conclusions about the implications of the CMS hospital price transparency rule for commercially insured patients using hospital care in the US.

## Conclusions

This cross-sectional study found that independent practitioners who billed for their services separately were frequently involved in the delivery of shoppable hospital-based care. In some encounters, such as most laboratory and pathology services, the undisclosed reimbursements of independent practitioners were relatively low; thus, the disclosed hospital prices could be beneficial to consumers. In other encounters, such as some evaluation and management services, radiology services, and most medicine and surgery services, the reimbursement of independent practitioners was often nontrivial and could create a substantial financial burden for patients who may not expect to receive additional bills beyond those from the hospital. The CMS hospital price transparency rule has recently begun requiring hospitals to publicly disclose plan-specific negotiated rates for a set of shoppable health care procedures and ancillary services provided to patients by hospital-employed practitioners. However, services integral to hospital-based care may be delivered and billed separately by independent practitioners or health care entities, which are not subject to this regulation. As such, health care price transparency remains an elusive goal that will require further innovation.

## Notes

Supplement. **eTable 1.** List of Ancillary Services that Could be Provided and Billed in Connection to the Primary Shoppable Health Care Service **eFigure 1.** Flowchart of Sample Inclusion and Exclusion Criteria **eTable 2.** Flowchart of Sample Inclusion and Exclusion Criteria by Service **eFigure 2.** Total Reimbursement for Service Packages Ranked by the Portion of Reimbursement Billed by the Hospital for Selected Common Shoppable Health Care Services
