# Supplementary material for: Concordance of Disclosed Hospital Prices With Total Reimbursements for Hospital-Based Care Among Commercially Insured Patients in the US
Source: JAMA Netw Open. 2021 Dec 13;4(12):e2137390. doi: 10.1001/jamanetworkopen.2021.37390 (PMC8669520; doi:10.1001/jamanetworkopen.2021.37390)
Supplement: Supplement. — eTable 1. List of Ancillary Services that Could be Provided and Billed in Connection to the Primary Shoppable Health Care Service eFigure 1. Flowchart of Sample Inclusion and Exclusion Criteria eTable 2. Flowchart of Sample Inclusion and Exclusion Criteria by Service eFigure 2. Total Reimbursement for Service Packages Ranked by the Portion of Reimbursement Billed by the Hospital for Selected Common Shoppable Health Care Services [file jamanetwopen-e2137390-s001.pdf]

Supplemental Online Content

Horný M, Shafer PR, Dusetzina SB. Concordance of disclosed hospital prices with total reimbursements for hospital-based care among commercially insured patients in the US. *JAMA Netw Open*. 2021;4(12):e2137390. doi:10.1001/jamanetworkopen.2021.37390

eTable 1. List of Ancillary Services that Could be Provided and Billed in Connection to the Primary Shoppable Health Care Service
eFigure 1. Flowchart of Sample Inclusion and Exclusion Criteria
eTable 2. Flowchart of Sample Inclusion and Exclusion Criteria by Service
eFigure 2. Total Reimbursement for Service Packages Ranked by the Portion of Reimbursement Billed by the Hospital for Selected Common Shoppable Health Care Services

This supplemental material has been provided by the authors to give readers additional information about their work.

© 2021 Horný M et al. *JAMA Network Open.*

eTable 1. List of Ancillary Services that Could be Provided and Billed in Connection to the Primary Shoppable Health Care Service

Primary shoppable service	Setting	Ancillary service included in package if billed together with the primary shoppable service	Procedure code	Service-specific inclusion criteria	Package-specific exclusion criteria
EVALUATION & MANAGEMENT					
Psychotherapy, 30/45/60 min (CPT 90832, 90834, 90837); Family psychotherapy, including/not including patient, 50 min (CPT 90846, 90847)	Outpatient	Facility fee			
		Psychiatric diagnostic evaluation	90791-2		
		Office or other outpatient visit for the evaluation and management of a new patient	99201-5	Only if billed by a psychiatrist	
		Office or other outpatient visit for the evaluation and management of an established patient	99211-5	Only if billed by a psychiatrist	
		Drug test(s), presumptive	80305-7, G0477-9		
		Drug test(s), definitive	G0480-3		
Group psychotherapy (CPT 90853)	Outpatient	** Same set of services as for Psychotherapy, 30/45/60 min, Family psychotherapy, including/not including patient, 50 min, with addition of:*			
		Group psychotherapy other than of a multiple-family group, in a partial hospitalization setting, approximately 45 to 50 minutes	G0410		
New patient office or other outpatient visit, typically 30/45/60 min (CPT 99203, 99204, 99205)	Outpatient	Facility fee			
		Hospital outpatient clinic visit for assessment and management of a patient	G0463		
		** "New patient office or other outpatient visit" is a broad health service that can be accompanied by various other procedures and services depending on the purpose of the visit.*			
Patient office consultation, typically 40/60 min (CPT 99243, 99244)	Outpatient	Facility fee			
Initial new patient preventive medicine evaluation, 18-39 years (CPT 99385)	Outpatient	Facility fee			
		Collection of venous blood by venipuncture	36415		
		Collection of capillary blood specimen	36416		
		Laboratory & pathology services	8*xxxx*		
		Immunization administration	90471-4		
		Vaccines	90476-749		
		Electrocardiogram, routine ECG	93000-5, G0403-5		
		Brief emotional/behavioral assessment	96127		
		Handling and/or conveyance of specimen for transfer to a laboratory	99000-1		

© 2021 Horný M et al. *JAMA Network Open.*

Primary shoppable service	Setting	Ancillary service included in package if billed together with the primary shoppable service	Procedure code	Service-specific inclusion criteria	Package-specific exclusion criteria
		Administration of influenza virus/pneumococcal/hepatitis B vaccine	G0008-10		
		Cervical or vaginal cancer screening	G0101		
		Screening cytopathology	G0123-4, G0141-7		
		Annual alcohol misuse screening	G0442		
		Annual depression screening	G0444		
		Hospital outpatient clinic visit for assessment and management of a patient	G0463		
		HIV antigen/antibody screening	G0475		
		HPV for cervical cancer screening	G0476		
		Screening papanicolaou smear	Q0091		
Initial new patient preventive medicine evaluation, 40-64 years (CPT 99386)	Outpatient	** same set of services as for Initial new patient preventive medicine evaluation (18-39 years), with addition of:*			
		Prostate cancer screening	G0102-3		
**LABORATORY & PATHOLOGY**					
All proposed shoppable laboratory & pathology services (CPT 80048, 80053, 80055, 80061, 80069, 80076, 81000, 81001, 81002, 81003, 84153, 84154, 84443, 85025, 85027, 85610, 85730)	Outpatient	Facility fee		Only if service was delivered at a hospital that bills for a facility fee for laboratory & pathology services alone	Collection of venous blood by venipuncture not billed (e.g., blood was collected through a port – an indication of a complex health care encounter)
		Collection of venous blood by venipuncture	36415		
**RADIOLOGY**					
CT scan, head or brain, without contrast (CPT 70450); X-Ray, lower back, minimum four views (CPT 72110); Ultrasound of abdomen (CPT 76700); Abdominal ultrasound of pregnant	Outpatient	Facility fee		Only if service was delivered at a hospital that bills for a facility fee for radiology services alone	

© 2021 Horný M et al. *JAMA Network Open.*

Primary shoppable service	Setting	Ancillary service included in package if billed together with the primary shoppable service	Procedure code	Service-specific inclusion criteria	Package-specific exclusion criteria
uterus, greater or equal to 14 weeks 0 days, single or first fetus (CPT 76805); Ultrasound pelvis through vagina (CPT 76830); Mammography of one breast/both breasts (CPT 77065, 77066); Mammography, screening, bilateral (CPT 77067)					
MRI scan of brain before and after contrast (CPT 70553)	Outpatient	Facility fee		Only if service was delivered at a hospital that bills for a facility fee for radiology services alone	
		Anesthesia for non-invasive imaging	01922		
		Contrast material	A95*xx*, Q9958-67		
MRI scan of lower spinal canal (CPT 72148); MRI scan of leg joint (CPT 73721)	Outpatient	Facility fee		Only if service was delivered at a hospital that bills for a facility fee for radiology services alone	
		Anesthesia for non-invasive imaging	01922		
CT scan, pelvis, with contrast (CPT 72193); CT scan of abdomen and pelvis with contrast (CPT 74177)	Outpatient	Facility fee		Only if service was delivered at a hospital that bills for a facility fee for radiology services alone	
		Contrast material	Q9958-67		
**MEDICINE & SURGERY**					
Cardiac valve and other major cardiothoracic	Inpatient	*All services billed during inpatient stay*			

© 2021 Horný M et al. *JAMA Network Open.*

Primary shoppable service	Setting	Ancillary service included in package if billed together with the primary shoppable service	Procedure code	Service-specific inclusion criteria	Package-specific exclusion criteria
procedures with cardiac catheterization with major complications or comorbidities (DRG 216)					
Spinal fusion except cervical without major comorbid conditions or complications (DRG 460)	Inpatient	*All services billed during inpatient stay*			
Major joint replacement or reattachment of lower extremity without major comorbid conditions or complications (DRG 470)	Inpatient	*All services billed during inpatient stay*			
Cervical spinal fusion without comorbid conditions or major comorbid conditions or complications (DRG 473)	Inpatient	*All services billed during inpatient stay*			
Uterine and adnexa procedures for non-malignancy without comorbid conditions or major comorbid conditions or complications (DRG 743)	Inpatient	*All services billed during inpatient stay*			
	Outpatient	Facility fee			

© 2021 Horný M et al. *JAMA Network Open.*

Primary shoppable service	Setting	Ancillary service included in package if billed together with the primary shoppable service	Procedure code	Service-specific inclusion criteria	Package-specific exclusion criteria
Removal of 1 or more breast growth, open procedure (CPT 19120)		Anesthesia for procedures on the integumentary system on the extremities, anterior trunk and perineum	00400, 00402		
		Excision, benign lesion including margins, except skin tag (unless listed elsewhere), trunk, arms or legs	11400-6		
		Repair, intermediate, wounds of scalp, axillae, trunk and/or extremities; 2.6 cm to 7.5 cm	12032		
		Adjacent Tissue Transfer or Rearrangement	14000-21		
		Placement of breast localization device(s), percutaneous; first lesion	19281, 19285		
		Revision of reconstructed breast	19380		
		Collection of venous blood by venipuncture	36415		
		Biopsy or excision of lymph node(s); open deep axillary node(s)	38525		
		Injection procedure; lymphangiography; radioactive tracer for identification of sentinel node	38792		
		Intraoperative identification (eg, mapping) of sentinel lymph node(s) includes injection of non-radioactive dye, when performed	38900		
		Radiological examination, surgical specimen	76098		
		Ultrasound, breast, unilateral, real time with image documentation; limited	76642		
		Ultrasonic guidance, intraoperative	76998		
		Laboratory & pathology services	8*xxxx*		
		Electrocardiogram, routine ECG; tracing only	93005		
		Injection, bupivacaine liposome	C9290		
		Drugs administered other than oral method	J*xxxx*		
		Injection, non-radioactive, non-contrast, visualization adjunct	Q9968		
		Injection, bupivicaine hydrochloride	S0020		
Shaving of shoulder bone using an arthroscope (CPT 29826)	Outpatient	Facility fee			
		Anesthesia for all procedures on nerves, muscles, tendons, fascia, and bursae of shoulder and axilla	01610		

© 2021 Horný M et al. *JAMA Network Open.*

Primary shoppable service	Setting	Ancillary service included in package if billed together with the primary shoppable service	Procedure code	Service-specific inclusion criteria	Package-specific exclusion criteria
		Anesthesia for diagnostic arthroscopic procedures of shoulder joint	01622		
		Anesthesia for open or surgical arthroscopic procedures on humeral head and neck, sternoclavicular joint, acromioclavicular joint, and shoulder joint	01630		
		Arthroscopy, shoulder, surgical	29806-25, 27, 28		
		Collection of venous blood by venipuncture	36415		
		Injection, anesthetic agent; brachial plexus, single	64415		
		Injection, anesthetic agent; trigeminal nerve; brachial plexus, continuous infusion by catheter	64416		
		Ultrasonic guidance for needle placement (eg, biopsy, aspiration, injection, localization device), imaging supervision and interpretation	76942		
		Laboratory & pathology services	8*xxxx*		
		Electrocardiogram, routine ECG; tracing only	93005		
		Anchor/screw for opposing bone-to-bone or soft tissue-to-bone (implantable);	C1713		
		Injection, bupivacaine liposome	C9290		
		Drugs administered other than oral method	J*xxxx*		
		Shoulder orthosis, prefabricated, off-the-shelf	L3650-78		
		Shoulder elbow wrist hand orthosis, abduction positioning, airplane design, prefabricated, includes fitting and adjustment	L3960		
		Injection, bupivicaine hydrochloride	S0020		
Removal of one knee cartilage using an endoscope (CPT 29881)	Outpatient	Facility fee			
		Anesthesia for all procedures on nerves, muscles, tendons, fascia, and bursae of shoulder and axilla	01610		
		Anesthesia for diagnostic arthroscopic procedures of shoulder joint	01622		
		Anesthesia for open or surgical arthroscopic procedures on humeral head and neck, sternoclavicular joint, acromioclavicular joint, and shoulder joint	01630		

© 2021 Horný M et al. *JAMA Network Open.*

Primary shoppable service	Setting	Ancillary service included in package if billed together with the primary shoppable service	Procedure code	Service-specific inclusion criteria	Package-specific exclusion criteria
		Arthroscopy, shoulder, surgical	29806-25, 27, 28		
		Collection of venous blood by venipuncture	36415		
		Injection, anesthetic agent; brachial plexus, single	64415		
		Injection, anesthetic agent; trigeminal nerve; brachial plexus, continuous infusion by catheter	64416		
		Ultrasonic guidance for needle placement (eg, biopsy, aspiration, injection, localization device), imaging supervision and interpretation	76942		
		Laboratory & pathology services	8*xxxx*		
		Electrocardiogram, routine ECG; tracing only	93005		
		Anchor/screw for opposing bone-to-bone or soft tissue-to-bone (implantable);	C1713		
		Injection, bupivacaine liposome	C9290		
		Drugs administered other than oral method	J*xxxx*		
		Shoulder orthosis, prefabricated, off-the-shelf	L3650-78		
		Shoulder elbow wrist hand orthosis, abduction positioning, airplane design, prefabricated, includes fitting and adjustment	L3960		
		Injection, bupivicaine hydrochloride	S0020		
Removal of tonsils and adenoid glands patient younger than age 12 (CPT 42820)	Outpatient	Facility fee			
		Anesthesia for intraoral procedures, including biopsy	00170		
		Ablation, soft tissue of inferior turbinates, unilateral or bilateral, any method; intramural (i.e., submucosal)	30802		
		Collection of venous blood by venipuncture	36415		
		Laboratory & pathology services	8*xxxx*		
		Initial observation care, per day	99218-20		
		Surgical supply; miscellaneous	A4649		
		Hospital observation	G0378		
		Drugs administered other than oral method	J*xxxx*		
Diagnostic examination of esophagus, stomach, and/or upper small	Outpatient	Facility fee			
		Anesthesia for procedures on the upper abdomen	00731		
		Laboratory & pathology services	8*xxxx*		

© 2021 Horný M et al. *JAMA Network Open.*

Primary shoppable service	Setting	Ancillary service included in package if billed together with the primary shoppable service	Procedure code	Service-specific inclusion criteria	Package-specific exclusion criteria
bowel using an endoscope (CPT 43235)		Esophagus, gastroesophageal reflux test; with mucosal attached telemetry pH electrode placement	91035		
		Moderate sedation services	99152		
		Moderate sedation services	G0500		
		Drugs administered other than oral method	J*xxxx*		
Biopsy of the esophagus, stomach, and/or upper small bowel using an endoscope (CPT 43239)	Outpatient	** same set of services as for Diagnostic examination of esophagus, stomach, and/or upper small bowel using an endoscope, with addition of:*			
		Diagnostic examination of esophagus, stomach, and/or upper small bowel using an endoscope	43235		
Diagnostic examination of large bowel using an endoscope (CPT 45378)	Outpatient	Facility fee			
		Anesthesia for procedures on the lower abdomen	00811-3		
		Anesthesia for anorectal procedure	00902		
		Laboratory & pathology services	8*xxxx*		
		Moderate sedation services	99152		
		Moderate sedation services	G0500		
		Drugs administered other than oral method	J*xxxx*		
Biopsy of large bowel using an endoscope (CPT 45380)	Outpatient	** same set of services as for Diagnostic examination of large bowel using an endoscope, with addition of:*			
		Diagnostic examination of large bowel using an endoscope	45378		
Removal of polyps or growths of large bowel using an endoscope (CPT 45385)	Outpatient	** same set of services as for Biopsy of large bowel using an endoscope*			
Ultrasound examination of lower large bowel using an endoscope (CPT 45391)	Outpatient	Facility fee			
		Anesthesia for procedures on the lower abdomen	00811-3		
		Anesthesia for anorectal procedure	00902		
		Moderate sedation services	99152		
		Moderate sedation services	G0500		
		Drugs administered other than oral method	J*xxxx*		

© 2021 Horný M et al. *JAMA Network Open.*

Primary shoppable service	Setting	Ancillary service included in package <u>if billed</u> together with the primary shoppable service	Procedure code	Service-specific inclusion criteria	Package-specific exclusion criteria
Removal of gallbladder using an endoscope (CPT 47562)	Inpatient	*All services billed during inpatient stay*			DRG other than 419 – Laparoscopic cholecystectomy without common bile duct exploration without complication, comorbidity or major complication or comorbidity
	Outpatient	Anesthesia for intraperitoneal procedures in upper abdomen including laparoscopy	00790		
		Collection of venous blood by venipuncture	36415		
		Transversus abdominis plane (TAP) block (abdominal plane block, rectus sheath block) bilateral; by injections	64488		
		Ultrasound, abdominal, real time with image documentation; limited	76705		
		Laboratory & pathology services	8*xxxx*		
		Electrocardiogram, routine ECG; tracing only	93005		
		Intravenous infusion, hydration	96360-1		
		Intravenous infusion, for therapy, prophylaxis, or diagnosis	96365-8		
		Intravenous push, single or initial substance/drug	96374-6		
		Initial observation care, per day	99218-20		
		Injection, pantoprazole sodium	C9113		
		Injection, bupivacaine liposome	C9290		
		Hospital observation	G0378		
		Drugs administered other than oral method	J*xxxx*		
		Injection, bupivicaine hydrochloride	S0020		
		Injection, famotidine	S0028		
		Surgical techniques requiring use of robotic surgical system	S2900		
	Outpatient	Facility fee			
		Anesthesia for hernia repairs in lower abdomen	00830		

© 2021 Horný M et al. *JAMA Network Open.*

Primary shoppable service	Setting	Ancillary service included in package <u>if billed</u> together with the primary shoppable service	Procedure code	Service-specific inclusion criteria	Package-specific exclusion criteria
Repair of groin hernia patient age 5 years or older (CPT 49505)		Anesthesia for intraperitoneal procedures in lower abdomen including laparoscopy	00840		
		Collection of venous blood by venipuncture	36415		
		Transversus abdominis plane (TAP) block (abdominal plane block, rectus sheath block) unilateral; by injection	64486		
		Transversus abdominis plane (TAP) block (abdominal plane block, rectus sheath block) bilateral; by injections	64488		
		Laboratory & pathology services	8*xxxx*		
		Electrocardiogram, routine ECG; tracing only	93005		
		Mesh (implantable)	C1781		
		Injection, bupivacaine liposome	C9290		
		Drugs administered other than oral method	J*xxxx*		
		Injection, bupivicaine hydrochloride	S0020		
Biopsy of prostate gland (CPT 55700)	Outpatient	Facility fee			
		Anesthesia for procedures on the integumentary system on the extremities, anterior trunk and perineum	00400		
		Anesthesia for; anorectal procedure	00902		
		Anesthesia for transurethral procedures	00910		
		Collection of venous blood by venipuncture	36415		
		Injection, anesthetic agent; other peripheral nerve or branch	64450		
		3D rendering with interpretation and reporting	76377		
		Ultrasound, transrectal	76872		
		Ultrasonic guidance for needle placement	76942		
		Ultrasonic guidance, intraoperative	76988		
		Laboratory & pathology services	8*xxxx*		
		Electrocardiogram, routine ECG; tracing only	93005		
		Therapeutic, prophylactic, or diagnostic injection; subcutaneous or intramuscular	96372		
		Moderate sedation services	99152		
		Surgical pathology, gross and microscopic examinations, for prostate needle biopsy, any method	G0416		

© 2021 Horný M et al. *JAMA Network Open.*

Primary shoppable service	Setting	Ancillary service included in package <u>if billed</u> together with the primary shoppable service	Procedure code	Service-specific inclusion criteria	Package-specific exclusion criteria
		Drugs administered other than oral method	J*xxxx*		
Surgical removal of prostate and surrounding lymph nodes using an endoscope (CPT 55866)	Inpatient	*All services billed during inpatient stay*			DRG other than 708 – Major male pelvic procedures without complication, comorbidity or major complication or comorbidity; or concurrent cystectomy: 51570-96 also billed
	Outpatient	Facility fee			Concurrent cystectomy: 51570-96 also billed
		Collection of venous blood by venipuncture	36415		
		Laparoscopy, surgical; with retroperitoneal lymph node sampling (biopsy), single or multiple	38570		
		Laparoscopy, surgical; with bilateral total pelvic lymphadenectomy	38571		
		Transversus abdominis plane (TAP) block (abdominal plane block, rectus sheath block) bilateral; by injections	64488		
		Laboratory & pathology services	8*xxxx*		
		Electrocardiogram, routine ECG; tracing only	93005		
		Noninvasive ear or pulse oximetry for oxygen saturation;	94760-1		
		Intravenous infusion, hydration	96360-1		
		Intravenous infusion, for therapy, prophylaxis, or diagnosis	96365-8		
		Intravenous push, single or initial substance/drug	96374-6		
		Initial observation care, per day	99218-20		
		Catheter, drainage	C1729		
		Catheter, ureteral	C1758		
		Closure device, vascular	C1760		
		Connective tissue, human	C1762		
		Guide wire	C1769		

© 2021 Horný M et al. *JAMA Network Open.*

Primary shoppable service	Setting	Ancillary service included in package if billed together with the primary shoppable service	Procedure code	Service-specific inclusion criteria	Package-specific exclusion criteria
		Mesh (implantable)	C1781		
		Port, indwelling (implantable)	C1788		
		Injection, pantoprazole sodium	C9113		
		Injection, bupivacaine liposome	C9290		
		Hospital observation	G0378		
		Drugs administered other than oral method	J*xxxx*		
		Infusion, albumin (human), 5%	P9045		
		Injection, bupivicaine hydrochloride	S0020		
		Surgical techniques requiring use of robotic surgical system	S2900		
		Amniotic membrane for surgical reconstruction	V2790		
Routine obstetric care for vaginal delivery, including pre-and post-delivery care (CPT 59400)	Inpatient	*All services billed during inpatient stay*			DRG other than 775 – Vaginal delivery without complicating diagnoses; or indication of cesarean delivery: 59510-622 also billed
Routine obstetric care for cesarean delivery, including pre-and post-delivery care (CPT 59510)	Inpatient	*All services billed during inpatient stay*			DRG other than 766 – Cesarean section without complication, comorbidity or major complication or comorbidity
Routine obstetric care for vaginal delivery after prior cesarean delivery including pre- and post-delivery care (CPT 59610)	Inpatient	*All services billed during inpatient stay*			DRG other than 775 – Vaginal delivery without complicating diagnoses; or indication of cesarean delivery: 59510-5 also billed
	Outpatient	Facility fee			

© 2021 Horný M et al. *JAMA Network Open.*

Primary shoppable service	Setting	Ancillary service included in package if billed together with the primary shoppable service	Procedure code	Service-specific inclusion criteria	Package-specific exclusion criteria
Injection of substance into spinal canal of lower back or sacrum using imaging guidance (CPT 62322, 62323)		Anesthesia for non-invasive imaging	01922		
		Anesthesia for percutaneous image guided procedures on the spine and spinal cord; therapeutic	01936		
		Radiologic examination, spine, single view	72020		
		Radiologic examination, spine, lumbosacral; 2 or 3 views	72100		
		Epidurography, radiological supervision and interpretation	72275		
		Fluoroscopic guidance and localization of needle or catheter tip for spine	77003		
		Moderate sedation services	99152		
		Office or other outpatient visit for the evaluation and management of a new patient	99201-5		
		Office or other outpatient visit for the evaluation and management of an established patient	99211-5		
		Catheter, intraspinal	C1755		
		Hospital outpatient clinic visit for assessment and management of a patient	G0463		
		Drugs administered other than oral method	J*xxxx*		
		Low osmolar contrast material	Q9965-7		
Injections of anesthetic and/or steroid drug into lower or sacral spine nerve root using imaging guidance (CPT 64483)	Outpatient	Facility fee			
		Anesthesia for percutaneous image guided procedures on the spine and spinal cord; therapeutic	01936		
		Anesthesia for diagnostic or therapeutic nerve blocks and injections, prone position	01992		
		Radiologic examination, spine, single view	72020		
		Radiologic examination, spine, lumbosacral; 2 or 3 views	72100		
		Epidurography, radiological supervision and interpretation	72275		
		Fluoroscopic guidance and localization of needle or catheter tip for spine	77003		
		Moderate sedation services	99152		

© 2021 Horný M et al. *JAMA Network Open.*

Primary shoppable service	Setting	Ancillary service included in package if billed together with the primary shoppable service	Procedure code	Service-specific inclusion criteria	Package-specific exclusion criteria
		Office or other outpatient visit for the evaluation and management of a new patient	99201-5		
		Office or other outpatient visit for the evaluation and management of an established patient	99211-5		
		Hospital outpatient clinic visit for assessment and management of a patient	G0463		
		Drugs administered other than oral method	J*xxxx*		
		Low osmolar contrast material	Q9965-7		
		Injection, bupivicaine hydrochloride	S0020		
Removal of recurring cataract in lens capsule using laser (CPT 66821)	Outpatient	Facility fee			
		Scanning computerized ophthalmic diagnostic imaging, posterior segment, with interpretation and report; optic nerve	92134		
		Ophthalmological services: medical examination and evaluation, with initiation or continuation of diagnostic and treatment program	92002, 92004, 92012, 92014		
		Office or other outpatient visit for the evaluation and management of an established patient	99211-5		
		Hospital outpatient clinic visit for assessment and management of a patient	G0463		
Removal of cataract with insertion of lens (CPT 66984)	Outpatient	Facility fee			
		Anesthesia for procedures on eye; lens surgery	00140-00145		
		Ophthalmic biometry by ultrasound echography; with intraocular lens power calculation	76519		
		Ophthalmic biometry by partial coherence interferometry with intraocular lens power calculation	92136		
		Lens, intraocular (new technology)	C1780		
		Ocular implant, aqueous drainage assist device	C1783		
		Drugs administered other than oral method	J*xxxx*		
		Posterior chamber intraocular lens	V2632		
		Astigmatism correcting function of intraocular lens	V2787		
		Presbyopia correcting function of intraocular lens	V2788		
Electrocardiogram, routine, with	Outpatient	Facility fee			

© 2021 Horný M et al. *JAMA Network Open.*

Primary shoppable service	Setting	Ancillary service included in package if billed together with the primary shoppable service	Procedure code	Service-specific inclusion criteria	Package-specific exclusion criteria
interpretation and report (CPT 93000)					
Insertion of catheter into left heart for diagnosis (CPT 93452)	Outpatient	Facility fee			
		Anesthesia for cardiac catheterization including coronary angiography and ventriculography	01920		
		Collection of venous blood by venipuncture	36415		
		Laboratory & pathology services	8*xxxx*		
		Electrocardiogram, routine ECG; tracing only	93005		
		Injection procedure during cardiac catheterization including imaging supervision; for supravalvular aortography	93567		
		Moderate sedation services	99152		
		Catheter, intravascular ultrasound	C1753		
		Drug wire	C1769		
		Catheter, guiding	C1887		
		Introducer/sheath, other than guiding, other than intracardiac electrophysiological	C1894		
		Percutaneous transcatheter placement of drug eluting intracoronary stent(s)	C9600		
		Hospital observation	G0378		
		Drugs administered other than oral method	J*xxxx*		
		Low osmolar contrast material	Q9967		
Sleep study (CPT 95810)	Outpatient	Facility fee			
		Multiple sleep latency or maintenance of wakefulness testing, recording, analysis and interpretation	95805		
		Office or other outpatient visit for the evaluation and management of a new patient	99201-5		
		Office or other outpatient visit for the evaluation and management of an established patient	99211-5		
		Hospital outpatient clinic visit for assessment and management of a patient	G0463		
Physical therapy, therapeutic exercise (CPT 97110)	Outpatient	Facility fee			
		Application of a modality to 1 or more areas; hot or cold packs	97010-6		

© 2021 Horný M et al. *JAMA Network Open.*

Primary shoppable service	Setting	Ancillary service included in package if billed together with the primary shoppable service	Procedure code	Service-specific inclusion criteria	Package-specific exclusion criteria
		Application of a modality to 1 or more areas; ultrasound	97035		
		Neuromuscular reeducation of movement, balance, coordination, kinesthetic sense, posture, and/or proprioception for sitting and/or standing activities	97112		
		Manual therapy techniques	97140		
		Physical therapy evaluation	97161-2		
		Therapeutic activities, direct (one-on-one) patient contact	97530		
		Electrical stimulation (unattended), to one or more areas for indication(s) other than wound care, as part of a therapy plan of care	G0283		

Notes: CPT, Current Procedural Terminology; DRG, Diagnosis-related group.

© 2021 Horný M et al. *JAMA Network Open.*

eFigure 1. Flowchart of Sample Inclusion and Exclusion Criteria

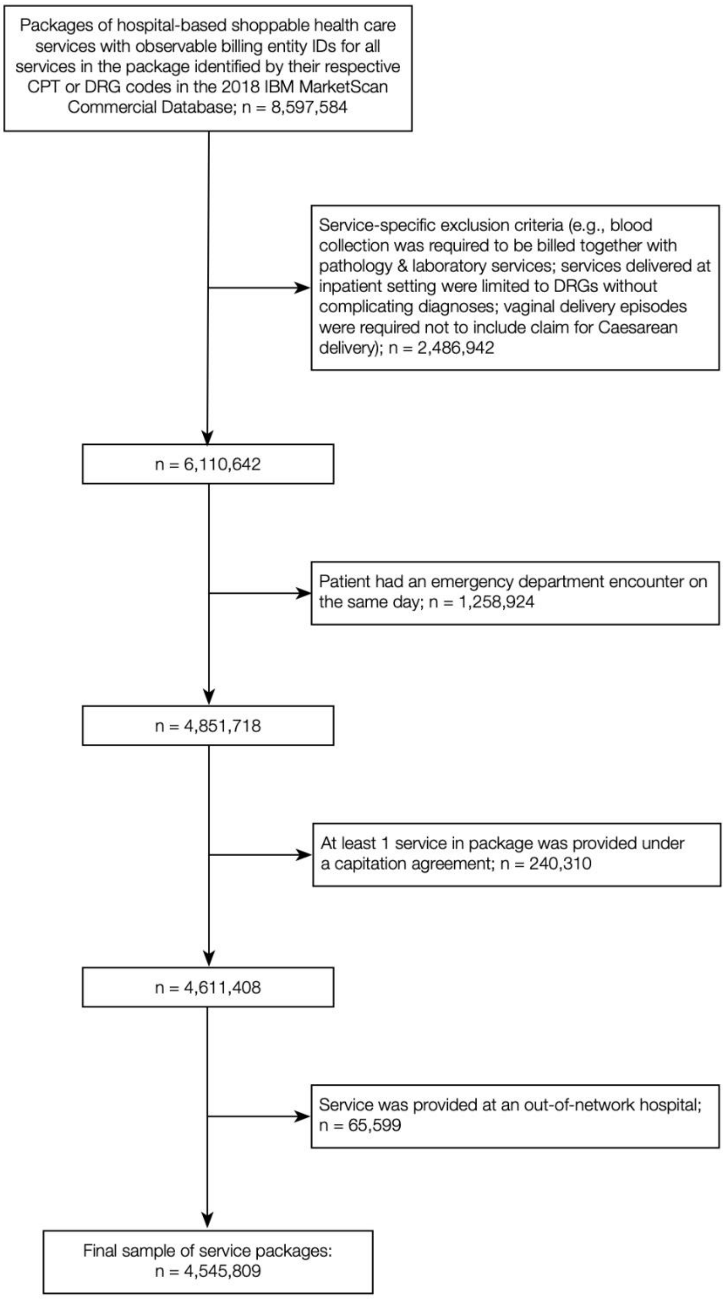

Notes: All 70 "shoppable" services proposed by the Centers for Medicare & Medicaid Services combined.

© 2021 Horný M et al. *JAMA Network Open.*

eTable 2. Flowchart of Sample Inclusion and Exclusion Criteria by Service

	Sample size				
**Shoppable service**	**(1) Hospital-based care with observable billing entity IDs**	**(2) Package-specific exclusions ***	**(3) Non-emergency**	**(4) Non-capitated**	**(5) In-network hospital**
**EVALUATION & MANAGEMENT**					
Psychotherapy, 30 min	9,018	9,018	8,952	8,111	7,126
Psychotherapy, 45 min	44,331	44,331	44,214	42,097	37,475
Psychotherapy, 60 min	18,427	18,427	18,369	17,229	14,940
Family psychotherapy, not including patient, 50 min	1,093	1,093	1,085	1,056	849
Family psychotherapy, including patient, 50 min	4,839	4,839	4,819	4,641	4,074
Group psychotherapy	85,196	85,196	84,962	80,983	69,840
New patient office or other outpatient visit, typically 30 min	15,585	15,585	14,503	12,563	12,038
New patient office of other outpatient visit, typically 45 min	10,786	10,786	10,310	8,516	8,204
New patient office of other outpatient visit, typically 60 min	4,483	4,483	4,280	3,842	3,682
Patient office consultation, typically 40 min	1,400	1,400	1,385	1,325	1,204
Patient office consultation, typically 60 min	1,503	1,503	1,490	1,421	1,365
Initial new patient preventive medicine evaluation (18-39 years)	960	960	959	913	885
Initial new patient preventive medicine evaluation (40-64 years)	655	655	654	632	605
**LABORATORY & PATHOLOGY**					
Basic metabolic panel	488,178	272,043	170,540	156,048	154,801
Blood test, comprehensive group of blood chemicals	1,285,020	799,610	582,264	547,507	542,383
Obstetric blood test panel	2,509	2,019	2,002	1,906	1,880
Blood test, lipids (cholesterol and triglycerides)	553,780	415,667	402,910	384,367	381,804
Kidney function panel test	20,357	13,768	12,582	11,844	11,666
Liver function blood test panel	138,358	73,727	46,879	44,333	43,972

© 2021 Horný M et al. *JAMA Network Open.*

	Sample size				
**Shoppable service**	**(1) Hospital-based care with observable billing entity IDs**	**(2) Package-specific exclusions ***	**(3) Non-emergency**	**(4) Non-capitated**	**(5) In-network hospital**
Manual urinalysis test with examination using microscope	444,413	192,361	87,567	80,264	79,415
Automated urinalysis test	278,635	108,014	48,115	42,677	42,215
PSA (prostate specific antigen)	79,909	56,902	56,659	54,232	53,810
Blood test, thyroid stimulating hormone (TSH)	492,189	359,758	332,483	315,369	313,096
Complete blood cell count, with differential white blood cells, automated	1,433,785	856,990	557,362	521,210	515,839
Complete blood count, automated	411,063	221,654	179,949	165,433	164,160
Blood test, clotting time	288,222	147,855	86,624	77,005	76,115
Coagulation assessment blood test	157,760	87,887	42,879	37,036	36,616
**RADIOLOGY**					
CT scan, head or brain, without contrast	112,963	112,963	9,635	9,322	9,144
MRI scan of brain before and after contrast	43,808	43,808	38,887	38,258	37,334
X-Ray, lower back, minimum four views	20,610	20,610	17,197	16,741	16,570
MRI scan of lower spinal canal	31,135	31,135	28,802	28,204	27,892
CT scan, pelvis, with contrast	1,795	1,795	896	870	832
MRI scan of leg joint	36,556	36,556	36,150	35,570	35,210
CT scan of abdomen and pelvis with contrast	147,238	147,238	51,028	49,837	47,555
Ultrasound of abdomen	41,870	41,870	35,641	34,794	34,430
Abdominal ultrasound of pregnant uterus (greater or equal to 14 weeks 0 days) single or first fetus	19,375	19,375	17,460	17,044	16,724
Ultrasound pelvis through vagina	70,085	70,085	51,518	50,339	49,759
Mammography of one breast	62,256	62,256	62,146	58,500	57,892
Mammography of both breasts	52,260	52,260	52,168	50,875	50,226
Mammography, screening, bilateral	431,493	431,493	430,836	421,853	418,328
**MEDICINE & SURGERY**					
Cardiac valve and other major cardiothoracic	110	110	23	23	22

© 2021 Horný M et al. *JAMA Network Open.*

	Sample size				
**Shoppable service**	**(1) Hospital-based care with observable billing entity IDs**	**(2) Package-specific exclusions ***	**(3) Non-emergency**	**(4) Non-capitated**	**(5) In-network hospital**
procedures with cardiac catheterization with major complications or comorbidities					
Spinal fusion except cervical without major comorbid conditions or complications	2,273	2,273	2,152	2,132	2,072
Major joint replacement or reattachment of lower extremity without major comorbid conditions or complications	12,910	12,910	12,716	12,556	12,257
Cervical spinal fusion without comorbid conditions or major comorbid conditions or complications	832	832	779	762	726
Uterine and adnexa procedures for non-malignancy without comorbid conditions or major comorbid conditions or complications	2,877	2,877	2,767	2,718	2,663
Removal of 1 or more breast growth, open procedure	1,663	1,663	1,653	1,633	1,611
Shaving of shoulder bone using an endoscope	4,506	4,506	4,482	4,428	4,376
Removal of one knee cartilage using an endoscope	7,381	7,381	7,351	7,090	6,974
Removal of tonsils and adenoid glands patient younger than age 12	4,268	4,268	4,237	4,182	4,131
Diagnostic examination of esophagus, stomach, and/or upper small bowel using an endoscope	7,316	7,316	6,784	6,556	6,403
Biopsy of the esophagus, stomach, and/or upper small bowel using an endoscope	48,766	48,766	46,924	45,607	44,596
Diagnostic examination of large bowel using an endoscope	28,100	28,100	27,833	27,453	26,889
Biopsy of large bowel using an endoscope	44,035	44,035	43,627	42,621	41,672

© 2021 Horný M et al. *JAMA Network Open.*

	Sample size				
**Shoppable service**	**(1) Hospital-based care with observable billing entity IDs**	**(2) Package-specific exclusions ***	**(3) Non-emergency**	**(4) Non-capitated**	**(5) In-network hospital**
Removal of polyps or growths of large bowel using an endoscope	28,419	28,419	28,224	27,594	27,131
Ultrasound examination of lower large bowel using an endoscope	53	53	53	50	43
Removal of gallbladder using an endoscope	12,715	10,984	8,717	8,623	8,487
Repair of groin hernia patient age 5 years or older	2,631	2,631	2,609	2,578	2,546
Biopsy of prostate gland	1,366	1,366	1,356	1,349	1,277
Surgical removal of prostate and surrounding lymph nodes using an endoscope	1,365	1,110	1,105	1,099	1,046
Routine obstetric care for vaginal delivery, including pre-and post-delivery care	46,616	38,615	33,611	33,316	32,753
Routine obstetric care for cesarean delivery, including pre-and post-delivery care	23,470	12,656	11,706	11,592	11,391
Routine obstetric care for vaginal delivery after prior cesarean delivery including pre-and post-delivery care	1,203	985	834	828	815
Injection of substance into spinal canal of lower back or sacrum using imaging guidance	8,545	8,545	8,452	8,249	8,076
Injections of anesthetic and/or steroid drug into lower or sacral spine nerve root using imaging guidance	8,986	8,986	8,922	8,643	8,384
Removal of recurring cataract in lens capsule using laser	450	450	450	440	433
Removal of cataract with insertion of lens	5,716	5,716	5,707	5,634	5,544
Electrocardiogram, routine, with interpretation and report	5,459	5,459	1,709	1,555	1,495
Insertion of catheter into left heart for diagnosis	57	57	48	46	45
Sleep study	13,153	13,153	13,138	12,888	12,768

© 2021 Horný M et al. *JAMA Network Open.*

	Sample size				
**Shoppable service**	**(1) Hospital-based care with observable billing entity IDs**	**(2) Package-specific exclusions ***	**(3) Non-emergency**	**(4) Non-capitated**	**(5) In-network hospital**
Physical therapy, therapeutic exercise	928,445	928,445	926,588	894,396	887,228

Notes: Package-specific exclusions are described in eTable 1.

© 2021 Horný M et al. *JAMA Network Open.*

eFigure 2. Total Reimbursement for Service Packages Ranked by the Portion of Reimbursement Billed by the Hospital for Selected Common Shoppable Health Care Services

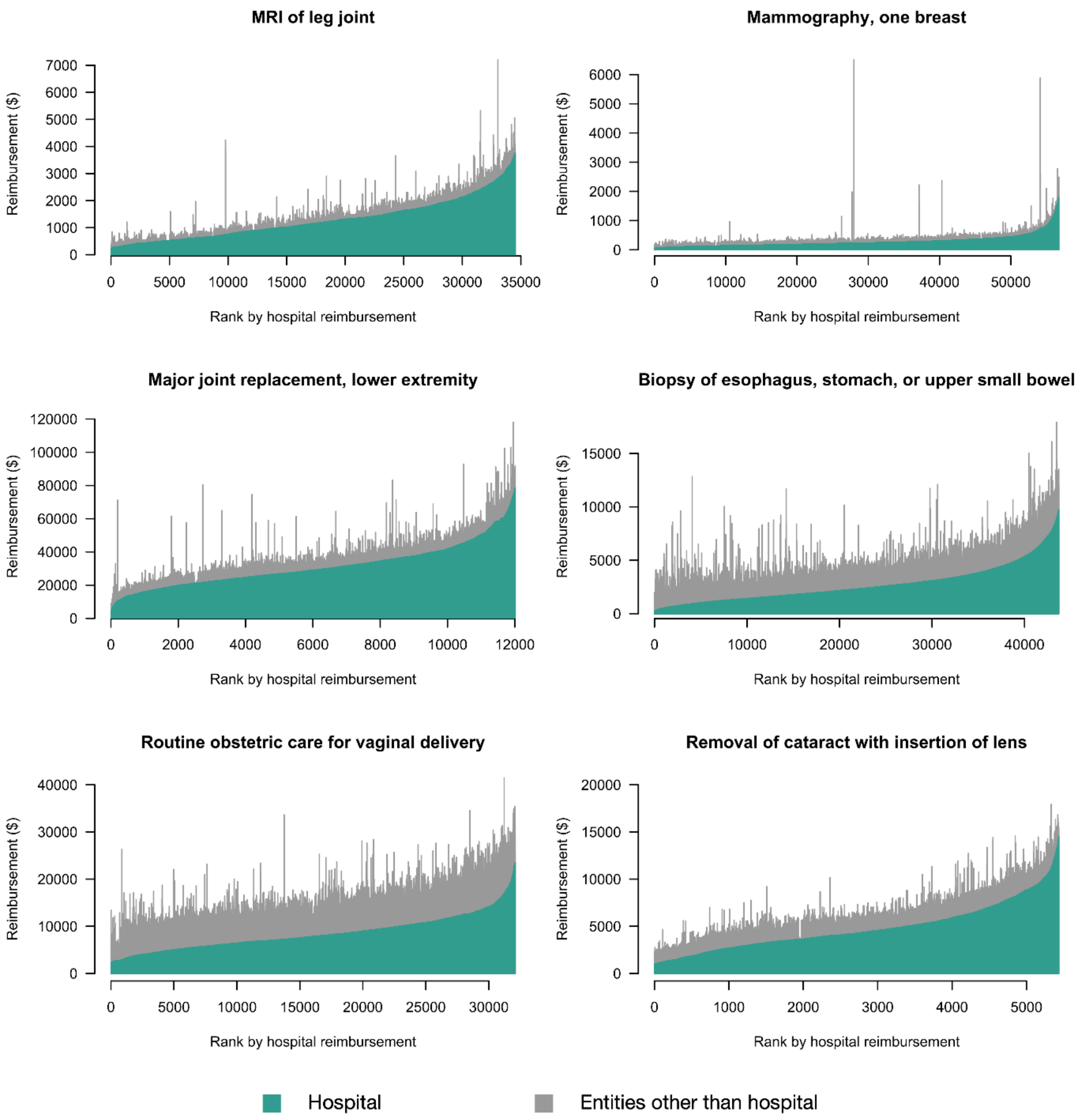

Notes: For better image quality, service packages with hospital reimbursement in the 1st and 99th percentiles were excluded from the visualizations.

© 2021 Horný M et al. *JAMA Network Open.*
